# MicroRNA Expression in Human Omental and Subcutaneous Adipose Tissue

**DOI:** 10.1371/journal.pone.0004699

**Published:** 2009-03-04

**Authors:** Nora Klöting, Susan Berthold, Peter Kovacs, Michael R. Schön, Mathias Fasshauer, Karen Ruschke, Michael Stumvoll, Matthias Blüher

**Affiliations:** 1 Department of Medicine, University of Leipzig, Leipzig, Germany; 2 Junior Research Group N06, Interdisciplinary Centre for Clinical Research (IZKF), University of Leipzig, Leipzig, Germany; 3 Städtisches Klinikum Karlsruhe, Clinic of Visceral Surgery, Karlsruhe, Germany; University of Camerino, Italy

## Abstract

MicroRNAs (miRNAs) are small non-coding RNAs, that play important regulatory roles in a variety of biological processes, including development, differentiation, apoptosis, and metabolism. In mammals, miRNAs have been shown to modulate adipocyte differentiation. Therefore, we performed a global miRNA gene expression assay in different fat depots of overweight and obese individuals to investigate whether miRNA expression in human adipose tissue is fat-depot specific and associated with parameters of obesity and glucose metabolism. Paired samples of abdominal subcutaneous (SC) and intraabdominal omental adipose tissue were obtained from fifteen individuals with either normal glucose tolerance (NGT, n = 9) or newly diagnosed type 2 diabetes (T2D, n = 6). Expression of 155 miRNAs was carried out using the TaqMan®MicroRNA Assays Human Panel Early Access Kit (Applied Biosystems, Darmstadt, Germany). We identified expression of 106 (68%) miRNAs in human omental and SC adipose tissue. There was no miRNA exclusively expressed in either fat depot, suggesting common developmental origin of both fat depots. Sixteen miRNAs (4 in NGT, 12 in T2D group) showed a significant fat depot specific expression pattern. We identified significant correlations between the expression of miRNA-17-5p, -132, -99a, -134, 181a, -145, -197 and both adipose tissue morphology and key metabolic parameters, including visceral fat area, HbA_1c_, fasting plasma glucose, and circulating leptin, adiponectin, interleukin-6. In conclusion, microRNA expression differences may contribute to intrinsic differences between omental and subcutaneous adipose tissue. In addition, human adipose tissue miRNA expression correlates with adipocyte phenotype, parameters of obesity and glucose metabolism.

## Introduction

MicroRNAs (miRNAs) are small (21–25 nucleotides in length), non-coding RNAs, that play an important regulatory role in animals and plants by targeting mRNA transcripts for cleavage or translational repression [1]–[3]. More than 900 unique mature miRNAs have been identified across species and their expression levels vary among species and tissues [4]. Following the discovery of the first miRNA in 1993, the important roles of miRNAs in a variety of biological processes, including development, differentiation, apoptosis, lipid metabolism, and cancer have been discovered [5]. In mammals, miRNAs have been shown to modulate stem cell and adipocyte differentiation as well as insulin secretion [6]–[8]. Esau et al. [9] found that reducing miRNA-143 by transfecting antisense oligonucleotides inhibits adipocyte differentiation *in vitro*, suggesting that miRNAs may play a role in the regulation of key adipose tissue processes and could therefore represent novel targets for the treatment of obesity. In addition, miRNAs have been shown to regulate metabolic processes, which are associated with type 2 diabetes including insulin signaling and glucose homeostasis [6], [10].

To gain further insight into the association between miRNA expression in human adipose tissue and parameters of obesity, fat distribution and glucose metabolism, we performed a global miRNA gene expression array in paired omental and abdominal subcutaneous adipose tissue samples from fifteen overweight or obese individuals with either normal glucose tolerance or type 2 diabetes. We tested the hypotheses that miRNA expression in human adipose tissue is fat-depot specific and related to parameters of obesity, fat distribution, glucose metabolism and adipose tissue morphology.

## Materials and Methods

### Ethics Statement

The study was approved by the Ethics Committee of the University of Leipzig. All subjects gave written informed consent before taking part in the study.

### Subjects

Paired samples of abdominal subcutaneous and intraabdominal omental adipose tissue were obtained from fifteen Caucasian men (*n* = 8) and women (*n* = 7) who underwent open abdominal surgery for elective cholecystectomy or explorative laparotomy. The age ranged from 50 to 73 years and the body mass index from 25.4 to 38.1 kg/m^2^. In addition to 9 patients with normal glucose tolerance (NGT), we newly diagnosed 6 individuals with type 2 diabetes (T2D). All subjects had a stable weight with no fluctuations of more than 2% of the body weight for at least three months before surgery. Only individuals, which fulfilled the previously reported inclusion criteria have included in the study [11]–[13]. Samples of omental and subcutaneous adipose tissue were immediately frozen in liquid nitrogen after explantation. Body mass index (BMI) was calculated as weight divided by squared height. Waist and hip circumferences were measured and waist-to-hip ratio was calculated. Percentage body fat was measured by dual X-ray absorptiometry (DEXA). Abdominal visceral and subcutaneous fat areas were calculated by CT scans measurement (L4–L5) [11]–[13].

### Assays and euglycemic–hyperinsulinemic clamp

Oral glucose tolerance test (OGTT) was performed according to the criteria of the American Diabetes Association (ADA) [14]. Blood samples were taken after an overnight fast to determine glucose, insulin, lipids and other standard laboratory parameters. Plasma insulin was measured using the IMMULITE automated analyzer (Diagnostic Products, Los Angeles, CA, USA). Leptin was measured with an ELISA (Mediagnost, Reutlingen, Germany). Plasma adiponectin was quantified with an ELISA (AdipoGen; Inc.; Seoul, South Korea). Serum C-reactive protein (CRP; immunoturbidometric latex test) and lipid analysis were performed with the ModularAnalytics EVO System (Roche Diagnostics GmbH, Mannheim, Germany). A high-sensitivity ELISA was used for IL-6 measurement (Quantikine IL-6, R&D Systems, Oxford, UK). Insulin sensitivity was assessed with the euglycemic-hyperinsulinemic clamp method, as described previously [15], [16].

### Characterization of adipose tissue morphology

For adipocyte size distribution analyses, adipocytes were isolated by collagenase (1 mg/ml) digestion. Aliquots of adipocytes were fixed with osmic acid and counted in triplicate using Multisizer III (Beckman-Coulter, Hamburg, Germany). For morphology studies, tissue samples were fixed in 10% buffered formalin and imbedded in paraffin. Multiple sections were analyzed systematically with respect to adipocyte size, number and macrophage number as previously described [17]. In brief, five micrometer sections were mounted on glass slides, de-paraffinized in Xylol and stained for CD68 using anti-CD68 monoclonal mouse anti-human antibody (Dako, close PGM1 M0876, dilution 1∶100), using standard immunohistochemistry methods. Macrophages were identified in the adipose parenchyma (CD68 within blood vessels were excluded), when cytoplasmic staining for CD68 was present along with an identifiable mononuclear nucleus, and presented as the number per 100 adipocytes (% macrophages) or as number of cells per 12×400 fields, as indicated. Staining of the sections was performed with hematoxylin and eosin. Images were acquired using BX60 microscope (Olympus) and were analyzed using Image-Pro Plus 4.0 software.

### Analysis of human adipose tissue miRNA gene expression

Human miRNA gene expression was performed with the TaqMan®MicroRNA Assays Human Panel Early Access Kit (Applied Biosystems, Darmstadt, Germany). Fluorescence was detected on an ABI PRISM 7700 sequence detector (Applied Biosystems, Darmstadt, Germany) using FAM as fluorescent reporter. Total RNA was isolated from paired subcutaneous and omental adipose tissue samples using TRIzol (Life Technologies, Grand Island, NY). cDNA was reversely transcribed from 5 ng total RNA samples using specific miRNA stem-loop primers from the TaqMan®MicroRNA Assays Human Panel. PCR products were synthesized from cDNA samples using sequence-specific primers from the TaqMan®MicroRNA Assays Human Panel (Applied Biosystems, Darmstadt, Germany).

Using the comparative CT method, hsa-miR-16 served as endogenous controls to normalize the expression levels of miRNA target genes by correcting differences in the amount of cDNA loaded into PCR reactions. Three *Caenorhabditis elegans* miRNAs, cel-lin-4, cel-miR-2, and cel-miR-124, and *Arabidopsis* miRNA, ath-miR-159a, served as negative controls. These four microRNAs were not detectable in the presence of human total RNA samples.

### Statistical analyses

Data are shown as means±standard errors of the mean (SEM) unless stated otherwise. Before statistical analysis, non-normally distributed parameters were logarithmically transformed to approximate a normal distribution. The following statistical tests were used: paired Student's *t* test and Pearson's simple correlation. Linear relationships were assessed by least square regression analysis. Statistical analysis was performed using SPSS version 12.0 (Chicago, IL). *P* values<0.05 were considered to be statistically significant. Since multiple statistical tests have been applied we performed additional Bonferroni correction for multiple comparisons, after which a p-value<0.001 was considered statistically significant.

## Results

### Human adipose tissue miRNA expression

Anthropometric and metabolic characteristics of study participants are summarized in Table 1. Gene expression analysis of 155 miRNAs in human paired omental and SC adipose tissue samples revealed that 106 (68%) out of 155 miRNAs are detectable in both omental and SC fat samples. There was no miRNA exclusively expressed in either fat depot. However, we found significant miRNA expression differences between omental and SC adipose tissue (Table 2). Interestingly, the miRNA expression pattern was different in NGT as compared to T2D patients (Table 2). In addition, we found significantly higher expression of miR-17-5p, miR-132, miR-134 in omental fat of NGT compared to T2D, whereas the opposite pattern was found for miR-181a. In SC fat, expression of miR-27a, miR-30e, miR-140, miR-155, miR-210 was significantly higher and expression of miR-147 and miR-197 was lower in NGT as compared to the T2D group

**Table 1:** Anthropometric and metabolic characteristics of the study groups.

	NGT (n = 9)	T2D (n = 6)	*p*-Value
	Mean±SEM	Mean±SEM	
Age (years)	66±3.3	67±2.8	>0.05
BMI (kg/m^2^)	28.6±2.0	31.2±2.1	>0.05
WHR	0.98±0.3	1.2±0.3	>0.05
Body fat (%)	30±2.6	32±2.6	>0.05
Visceral fat area (cm^2^)	123±8.7	294±7.4	<0.001
Subcutaneous fat area (cm^2^)	396±19	387±13	>0.05
Visceral/ SC fat area ratio	0.59±0.5	0.87±0.5	<0.05
Fasting plasma insulin (pmol/l)	59±7.7	264±9.9	<0.001
Fasting plasma glucose (mmol/l)	5.4±0.6	7.7±0.9	<0.001
HbA_1c_ (%)	5.4±0.4	6.7±0.8	<0.001
Glucose infusion rate (µmol · kg^−1^ · min^−1^)	82±3.8	26±3.3	<0.001
Total cholesterol (mmol/l)	5.0±1	5.9±0.7	<0.05
HDL cholesterol (mmol/l)	1.0±0.5	1.1±0.4	>0.05
LDL cholesterol (mmol/l)	2.8±1.0	3.6±0.5	<0.05
Free fatty acids (mmol)	0.35±0.5	0.70±0.5	<0.01
IL-6 (pg/ml)	6.0±2.4	7.6±1.5	>0.05
Leptin (pg/ml)	28.1±4.5	29.4±3.8	>0.05
Adiponectin (pg/ml)	6.8±1.9	3.0±1.2	<0.01
hsCRP (mmol)	8.9±4	7.8±2.3	>0.05
Macrophages in omental fat (%)	4.5±1.9	14.0±1.4	<0.001
Macrophages in SC fat (%)	2.3±1.4	4.0±1	>0.05

**Table 2:** miRNA gene expression differences between omental and subcutaneous fat.

hsa-miRNA	Omental fat	Subcutaneous fat	n-fold change	*p*-Value
***NGT***	Mean±SEM	Mean±SEM	Mean±SEM	
miR- 20	9.4±0.7	8.3±1.0	1.13	0.03
miR -98	9.7±1.7	8.1±0.5	1.2	0.04
miR-197	4.3±0.6	3.6±0.4	1.2	0.04
miR-331	7.0±1.4	5.5±0.5	1.27	0.01
***T2D***				
miR-17-5p	7.4±0.9	5.8±1.0	1.27	0.02
miR-27a	7.0±1.4	4.9±1.4	1.43	0.03
miR-29b	9.3±2.4	5.5±2.0	1.7	0.01
miR-30c	5.4±1.9	2.9±1.2	1.86	0.03
miR-126	4.6±0.7	2.9±1.2	1.59	0.04
miR-140	7.1±0.9	4.7±1.1	1.51	0.007
miR-145	1.0±0.8	0.2±0.7	5.0	0.025
miR-182	15.5±0.9	12.8±0.9	1.21	0.005
miR-195	6.1±0.4	4.1±1.8	1.49	0.03
miR-199a	4.0±0.7	2.1±1.4	1.9	0.02
miR-199b	8.3±1.1	6.1±1.8	1.36	0.03
miR-335	7.7±0.7	5.5±2.2	1.4	0.04

### Expression of selected miRNAs in adipose tissue correlates with parameters of obesity, metabolism, and morphology of adipose tissue

We further tested the hypothesis that expression of miRNAs is related to adipose tissue and adipocyte phenotype, parameters of obesity and glucose metabolism by univariate regression analyses. We identified six miRNAs in omental (Table 3) and four miRNAs in SC fat, which are significantly associated with adipose tissue morphology, parameters of obesity, glucose and lipid metabolism. Visceral fat area was negatively associated with the expression of miR-17-5p and miR-132 (Table 3). In SC fat, BMI is significantly correlated with the expression of miR-34a (r = −0.71, p = 0.004) and miR-145 (r = 0.53, p<0.05). In addition to the significant correlations between metabolic and circulating parameters and expression of selected miRNAs in visceral adipose tissue (Table 3), we identified significant correlations between SC fat expression of miR-145, HDL-cholesterol (r = −0.63, p<0.01), and 2-hour OGTT glucose levels (r = 0.93, p<0.01). miR-197 expression in SC fat correlates with fasting plasma glucose (r = 0.7, p = 0.004) and glucose infusion rate during in the clamp (r = −0.77, p = 0.002). SC miR-95 expression is related to serum concentrations of adiponectin (r = 0.52, p<0.05), CrP (r = −0.59, p<0.05), and IL-6 (r = −0.53, p<0.05).

**Table 3:** Significant correlates of miRNA expression.

	miR17-5p	miR-132	miR-134	miR-181a	miR-99a	miR-325
***Body composition***	r (p-value)	r (p-value)	r (p-value)	r (p-value)	r (p-value)	r (p-value)
Visceral fat area	−0.580 (p<0.05)	−0.729 (p<0.01)				
***Glucose homeostasis***						
FPG		−0.751 (p<0.001)				
FPI		−0.624 (p<0.05)			−0.545 (p<0.05)	
GIR	0.624 (p<0.05)	0.642 (p<0.05)	0.655 (p<0.05)			
HbA_1c_	−0.66 (p<0.01)		−0.59 (p<0.05)	0.56 (p<0.05)		
***Lipids and hormones***						
Total Cholesterol		−0.68 (p<0.01)			−0.63 (p<0.01)	
HDL-Cholesterol					0.56 (p<0.05)	
LDL-Cholesterol		−0.59 (p<0.05)				
Free fatty acids					−0.62 (p<0.01)	
Leptin						−0.64 (p<0.01)
Adiponectin				−0.59 (p<0.05)		
IL-6					−0.56 (p<0.05)	−0.6 (p<0.05)

**Figure 1:**
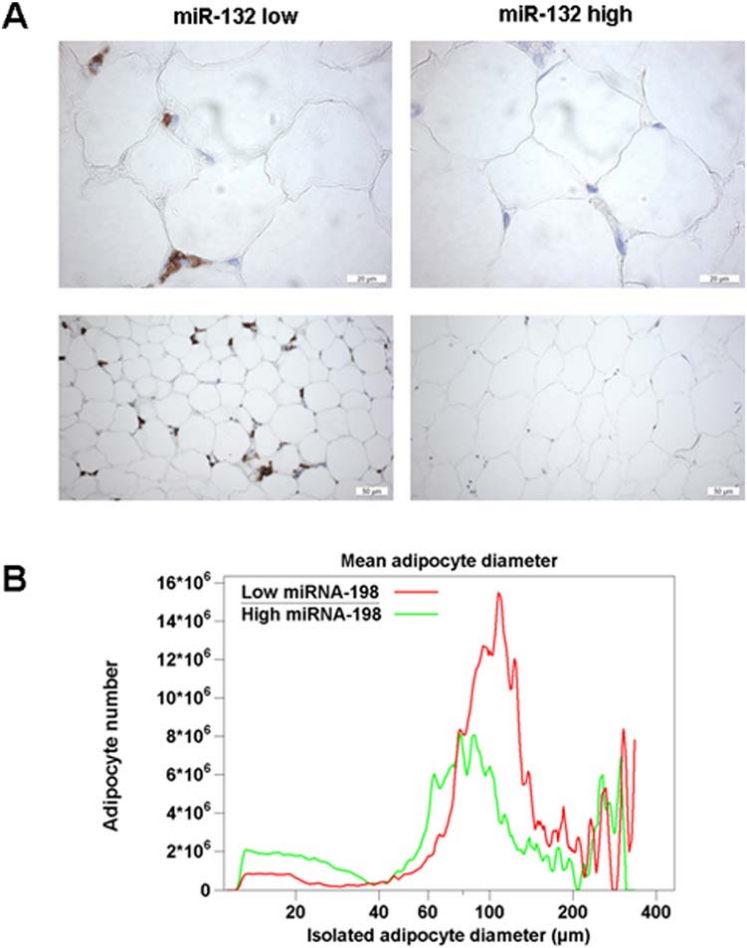
Relationships between miRNA (miR-132 and miR-198) expression and morphology of adipose tissue. (A) Typical example of omental adipose tissue morphology demonstrating lower macrophage infiltration in a patient with high miRNA-132 expression (miR-132 high) compared to a patient with low miRNA-132 expression (miR-132 low). Macrophage infiltration was assessed by immunostaining for CD68 as described in the methods. Upper slides, magnification 20×, lower slides, magnification 10× (B) Mean omental adipocyte diameter in a patient with high miRNA-198 expression (miR-198 high) compared to a patient with low miRNA-198 expression (miR-198 low). Aliquots of adipocytes were fixed with osmic acid and counted in triplicate using Multisizer III (Beckman-Coulter, Hamburg, Germany).

We identified 11 miRNAs representing 7% of all analyzed miRNAs, which are significantly related to the morphology of adipose tissue, including macrophage infiltration, mean and maximum adipocyte volume (Table 4). Among them, only miRNA-95 expression was found to be related to adipocyte size in both fat depots. Omental miRNA-132 (Figure 1A) and SC miRNA-26b and miRNA-155 expression are significantly related to the number of macrophages infiltrating the respective fat depot (Table 4). The strongest relationship between mean omental adipocyte diameter and microRNA expression was found for miRNA-198 (Figure 1B, Table 4).

**Table 4:** Relationships between miRNA expression and morphology of adipose tissue.

	Macrophage infiltration (%)	Mean adipocyte volume	Maximal adipocyte volume
***Omental fat***	r (p-value)	r (p-value)	r (p-value)
miRNA-19a		−0.75 (p<0.001)	
miRNA-95			−0.57 (p<0.05)
miRNA-132	−0.72 (p<0.01)		
miRNA-155		−0.58 (p<0.05)	
miRNA-198		−0.82 (p<0.001)	
***SC fat***			
miRNA-26b	−0.53 (p<0.05)		
miRNA-92			−0.55 (p<0.05)
miRNA-95		−0.53 (p<0.05)	−0.54 (p<0.05)
miRNA-155	−0.61 (p<0.05)		
miRNA-181a		−0.72 (p<0.01)	
miRNA-311		−0.78 (p<0.001)	

## Discussion

It has been recently suggested that miRNA expression contributes to the regulation of adipose tissue differentiation [9] metabolic processes linked to obesity and type 2 diabetes [6], [10]. A potential role of miRNAs in fat metabolism is supported by the finding in *Drosophila melanoaster* that microRNA-14 suppresses cell death and is required for normal fat metabolism [18]. It was shown that individual tissues maintain a unique miRNA profile, suggesting that miRNAs contribute to specific tissue function by regulating different gene targets [19]. Based on these findings and the known heterogeneity of different adipose tissue depots [20], we tested the hypotheses that miRNA expression in human adipose tissue is fat depot specific and related to parameters of adipose tissue morphology, obesity and glucose metabolism.

Here, we report that at least 106 different miRNAs are expressed in paired samples of human visceral and SC adipose tissue. Although we found significant expression differences in 16 miRNAs between the two depots, there was no miRNA exclusively expressed in either depot. This result suggests that visceral and SC adipose tissue are indeed two locations of a developmentally homogeneous adipose organ. We found significant miRNA expression differences between omental and SC fat depots. Most strikingly, all miRNA expression differences were due to significantly higher expression in omental fat (Table 2). Quantitative miRNA expression differences between the two fat depots could contribute to the well described differences in function and gene expression [20] between visceral and SC adipose tissue. On the other hand, factors including chronic hyperglycemia, altered adipokine serum profile in obesity and inflammation could alter miRNA expression in adipose tissue. Supporting this hypothesis, we found significant miRNA expression pattern differences between NGT and T2D patients. In addition, some miRNA levels significantly correlate with parameters of glucose metabolism, circulating marker of inflammation and adipokines.

Our data suggest that expression of miR-17-5p, miR-132, miR-134, miR-181a, miR-27a, miR-30e, miR-140, miR-147, miR-155, miR-197, and miR-210 play a role in the link between adipose tissue dysfunction and the development of obesity associated disorders including type 2 diabetes. A causal role of miR17-5p and miR-132 in obesity related impaired insulin sensitivity is suggested by the result that both miRNAs are significantly related to lower visceral fat mass, lower circulating parameters of chronic glycemia as well as improved insulin sensitivity. It needs to be tested, whether these correlations are due to altered expression of miR17-5p and miR-132 target genes, related to determination of visceral fat mass. In this context, there is recent evidence that miRNA-132 regulates expression of the cAMP response element-binding (CREB) protein, which plays a role in glucose homeostasis [21], [22]. Another miRNA we found to be significantly related to insulin sensitivity and chronic hyperglycemia is miR-134, which has been previously described as modulator of stem cell differentiation [7]. We therefore hypothesize that miR-134 plays a role in adipocyte differentiation and may contribute to the either hypertrophic insulin resistant or hyperplastic insulin sensitive subtype of obesity [23].

However, more sophisticated study designs are necessary to dissect causal from correlative relationships with miRNA expression in fat. Another key finding of this study is that ∼7% of the detectable miRNAs are significantly associated with adipocyte size, suggesting that these miRNAs are involved in the regulation of adipocyte development. Further studies are required to unravel the target genes of these miRNAs, which might be involved in determination of adipocyte size. Moreover, significant relationships between the expression of three miRNAs and the number of macrophages infiltrating adipose tissue suggests that miRNAs play a role in attracting immune cells to adipose tissue. Since we used adipose tissue samples, we can not dissect whether adipocytes or cells of the stromal vascular fraction cause fat depot specific miRNA expression differences and the described significant correlations with metabolic parameters. However, it is unlikely that increased macrophage infiltration itself causes a negative relationship with the expression of miR-132, -26b, and -155. Further studies will elucidate target mRNAs of these miRNAs, which might be causally related to macrophage infiltration into adipose tissue.

Noteworthy, our study has several limitations. Obviously, the number of subjects was relatively small and the described associations between anthropometric and metabolic parameters and miRNA expression will require confirmation in larger cohorts. We can also not exclude that specific characteristics of our study population, i.e. wide range of BMI and glucose tolerance distribution produced the described significant correlations. After appropriate adjustment for confounding variables and Bonferroni correction for multiple comparisons, only relationships with a p-value<0.001 could be considered statistically significant. However, raising the threshold for significant results by Bonferroni correction might lead to discarding some findings which could be biologically meaningful. To date there is no study on the relationship of microRNA expression in human subcutaneous and omental adipose tissue and therefore, we believe it is necessary to report our findings that may be verified by studies in larger cohorts. By showing unadjusted p-values, we follow the view of Kenneth J. Rothman that no adjustments are needed for multiple comparisons, if the unadjusted P-values are declared as such, so that the data can be exposed to verification by others [24]. In addition, there are some difficulties in the interpretation of miRNA expression data. For example, to investigate biological functions of miRNAs, it is critical to identify miRNA-directed target genes. Currently available computational methods (e.g., miRanda, PicTar, and TargetScan) predict numerous target genes that contain many false positives for miRNA [25], [26]. Also, experimental verification of miRNA-target relationship is complicated by the potential outcome of such an interaction being either translational repression or degradation. Furthermore, miRNAs can target multiple genes, and thereby the biological function of a single miRNA can be diverse. Hence, not only to achieve a higher degree of specificity of the prediction, but also to comprehensively understand the function of miRNA, large-scale prediction of targets across a whole genome would be required [24].

In conclusion, miRNA expression patterns in omental and SC adipose tissue suggest a common developmental origin of both fat depots. Differences in miRNA expression might contribute to differences in adipose tissue biology between omental and subcutaneous depots. In addition, human adipose tissue miRNA expression correlates with adipocyte phenotype, parameters of obesity and glucose metabolism.

### Statement

The funders had no role in study design, data collection and analysis, decision to publish, or preparation of the manuscript.
